# Genomic Comparison of *Salmonella* Enteritidis Strains Isolated from Laying Hens and Humans in the Abruzzi Region during 2018

**DOI:** 10.3390/pathogens9050349

**Published:** 2020-05-05

**Authors:** Lisa Di Marcantonio, Anna Janowicz, Katiuscia Zilli, Romina Romantini, Stefano Bilei, Daniela Paganico, Tiziana Persiani, Guido Di Donato, Elisabetta Di Giannatale

**Affiliations:** 1 Bacteriology and Diary Production Hygiene department, Istituto Zooprofilattico Sperimentale dell’Abruzzo e del Molise ‘G. Caporale’, Campo Boario, 64100 Teramo, Italy; a.janowicz@izs.it (A.J.); k.zilli@izs.it (K.Z.); r.romantini@izs.it (R.R.); d.paganico@izs.it (D.P.); t.persiani@izs.it (T.P.); e.digiannatale@izs.it (E.D.G.); 2 Food Microbiology department, Istituto Zooprofilattico Sperimentale del Lazio e della Toscana “M. Aleandri”, Via Appia Nuova 1411, 00178 Rome, Italy; stefano.bilei@izslt.it; 3 Epidemiology and Risk Analysis department, Istituto Zooprofilattico Sperimentale dell’Abruzzo e del Molise ‘G. Caporale’, Campo Boario, 64100 Teramo, Italy; guido.didonato@aslteramo.it

**Keywords:** *Salmonella* Enteritidis, salmonellosis, cgMLST, SNP address, epidemiology

## Abstract

Salmonellosis is a major cause of bacterial foodborne infection. Since 2016, an increased number of cases of gastroenteritis caused by *Salmonella enterica* serovar Enteritidis linked to eggs produced in Poland has been reported in Europe. In Italy, *S.* Enteritidis is one of the three most commonly reported serotypes, associated mainly with the consumption of contaminated eggs and derived products. In our work, we analysed 61 strains of *S.* Enteritidis obtained from humans and farms in the Abruzzi region, Italy, in 2018. We used Multiple-Loci Variable-Number Tandem Repeat (VNTR) analysis (MLVA)-based typing and Whole-Genome Sequencing (WGS) tools to identify closely related strains and perform cluster analysis. We found two clusters of genetically similar strains. The first one was present in the local farms and isolated from human cases and had single-linkage distance of no more than two core genes and less than five Single-Nucleotide Polymorphisms (SNPs). The second cluster contained strains isolated from humans and from a dessert (tiramisù) sample that shared identical core genome and were assigned the same SNP address. Cluster 2 isolates were found to be genetically similar to an *S.* Enteritidis strain from a multi-country outbreak linked to Polish eggs.

## 1. Introduction

Salmonellosis is the second most common gastrointestinal disease in Europe. In 2018, more than 90,000 confirmed salmonellosis cases were reported in the European Union, half of which were caused by *Salmonella enterica* serotype Enteritidis. The majority of these cases were linked to the consumption of eggs and egg-containing products, followed by that of bakery and mixed foods [1]. 

In Italy, 3635 cases of *Salmonella* infection were officially reported in 2018. More than 150 local human outbreaks were related to food consumption, and 34 were water-borne. In contrast to what observed in the EU, in Italy, Enteritidis was the third most common serovar isolated from patients after Typhimurium and a monophasic variant of *Salmonella* Typhimurium. An Italian national survey revealed that 0.8% of laying hens and less than 0.1% of broiler flocks tested positive for *S*. Enteritidis in the same year [1]. 

While salmonellosis in humans leads to gastroenteritis, cramps, diarrhoea, vomiting and is frequently accompanied by fever, in poultry, *S.* Enteritidis most commonly colonizes the cecum asymptomatically and can be shed in the faeces for prolonged periods. Occasionally, the pathogen escapes into the bloodstream of infected birds, leading to the infection of reproductive organs, which can result in the vertical transmission of *Salmonella* to eggs [2]. Consumption of food prepared with raw eggs contaminated with *Salmonella*, such as mayonnaise or desserts like tiramisù, represents a particularly common source of infection and frequently results in local outbreaks [3,4]. To reduce the risk of salmonellosis from liquid egg products, pasteurization is used. However, this process does not always lead to complete elimination of the pathogen [5,6]. Indeed, outbreaks of *S.* Enteritidis attributed to the consumption of chocolate mousse prepared using pasteurized egg whites have been reported in the past [7]. For decades, in most laboratories, outbreak investigations have been carried out through serotyping and phage typing, and to date, serotyping remains the standard routine technique for the analysis of *Salmonella* isolates [8]. However, serotyping and phage typing can only be used for the identification of potential small-scale outbreaks and require confirmation by other methods [9]. In the past, Pulsed-Field Gel Electrophoresis (PFGE) was used as a standard technique for outbreak investigations, but attempts have been made to replace it by Multiple-Loci Variable-Number Tandem Repeat (VNTR) analysis (MLVA), due to its simplicity and reproducibility [10,11,12]. This technique showed sufficient discriminative power in the analysis of distantly related strains and proved suitable for the purpose of surveillance of *S. enterica* serovars including *S.* Enteritidis and *S.* Typhimurium [13]. However, for in-depth outbreak investigations, it should be complemented by other typing methods [14]. 

In recent years, whole-genome sequencing (WGS)-based typing techniques, such as Single-Nucleotide Polymorphism (SNP) and core-genome/whole-genome Multilocus Sequence Typing (cgMLST/wgMLST) have been adopted by many laboratories [15,16,17,18]. WGS analysis offers the highest resolution for discriminating between genetically related and unrelated bacterial strains and is recommended by international bodies such as the European Food Standards Agency (EFSA) and the European Centre for Disease Prevention and Control (ECDC) for the analysis of foodborne outbreaks [19].

In 2016, an increase in salmonellosis cases due to *S*. Enteritidis reported in Scotland and in the Netherlands and caused by strains with uncommon multiple-locus variable-number tandem repeats led to a large-scale European investigation into possible sources of the multinational outbreak. Since then, the cases linked to eggs produced in Poland have been found in at least 15 countries, including Italy [19]. Indeed, Poland resulted to be one of the main eggs exporters in Europe. In the Abruzzi region, from 2012 to 2017, *S.* Enteritidis strains were rarely isolated from animal sources; however, in 2018, an increased prevalence in laying hens was observed. In fact, in 2018, 18 salmonellosis outbreaks in aviaries in several regions of Italy and caused by this *Salmonella* serovar were notified in the Information System of Animal Diseases (SIMAN). The outbreaks were reported in Abruzzi (6), Campania (1), Lazio (1), Lombardia (5), Molise (2), Apulia (1), Marche (1) and Veneto (1).

In 2018, multiple cases of human gastroenteritis caused by *S*. Enteritidis also occurred in the Abruzzi region of Italy. These included a group of people from another region who visited a local restaurant. The food samples from the restaurant were tested for presence of foodborne microorganisms, and *S.* Enteritidis was isolated from tiramisù. Other cases were not traced to specific food products and involved individual households unrelated to one another. The aim of this work was therefore to analyse *S.* Enteritidis strains isolated from patients and farms in Abruzzi, in order to understand if human infections could be attributed to locally circulating strains or were part of the large outbreak in the EU. We used the traditional MLVA approach and WGS tools to type the isolates and to identify potential outbreak clusters.

## 2. Results

**Figure 1:**
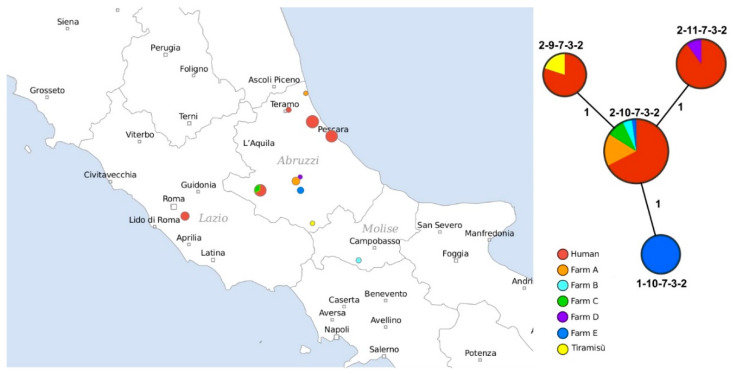
Distribution of *Salmonella* Enteritidis strains isolated in the regions of Abruzzi and Molise in 2018. The locations where the samples were isolated are shown on the map. Samples obtained from humans and from tiramisù are shown in red and yellow, respectively. Samples collected from different farms are indicated by different colours. Sample clustering according to Multiple-Loci Variable-Number Tandem Repeat analysis (MLVA) profiles is shown on the right.

Sixty-one isolates of *S.* Enteritidis were obtained during a surveillance and control programme of salmonellosis in Abruzzi region, Italy, in 2018 (Figure 1). Within this dataset, 42 strains were obtained from patients suffering from gastroenteritis, and 18 from poultry farms (either from chickens or from the farm environment). The set of human samples contained six strains that were isolated at the Istituto Zooprofilattico Lazio e Toscana in Rome from patients who had dined at the same restaurant in Abruzzi in a period prior to developing the symptoms of salmonellosis. *S.* Enteritidis strain was isolated from tiramisù that the infected persons had eaten. We tested the isolates for resistance to 14 antimicrobials. Except for one isolate that showed resistance to colistin, the strains were susceptible to all antimicrobials in the panel. We additionally typed the strains using the Multilocus Sequence Typing (MLST) method and found that all isolates belonged to ST-11.

Four different MLVA profiles were detected in the dataset (Figure 1), three of which were attributed to more than one strain. The isolates from humans matched three MLVA profiles: 2-9-7-3-2, 2-11-7-3-2 and 2-10-7-3-2. The majority of isolates from farms and patients showed the last profile. The genotypes of the six persons who ate at the same location, were split between two profiles, 2-9-7-3-2 and 2-10-7-3-2, the first of which was also assigned to the strain isolated from tiramisù. Interestingly, this MLVA profile was flagged by EFSA and ECDC for defining a “probable outbreak case” in the multi-country outbreak of *S.* Enteritidis linked to Polish eggs [19]. 

**Figure 2:**
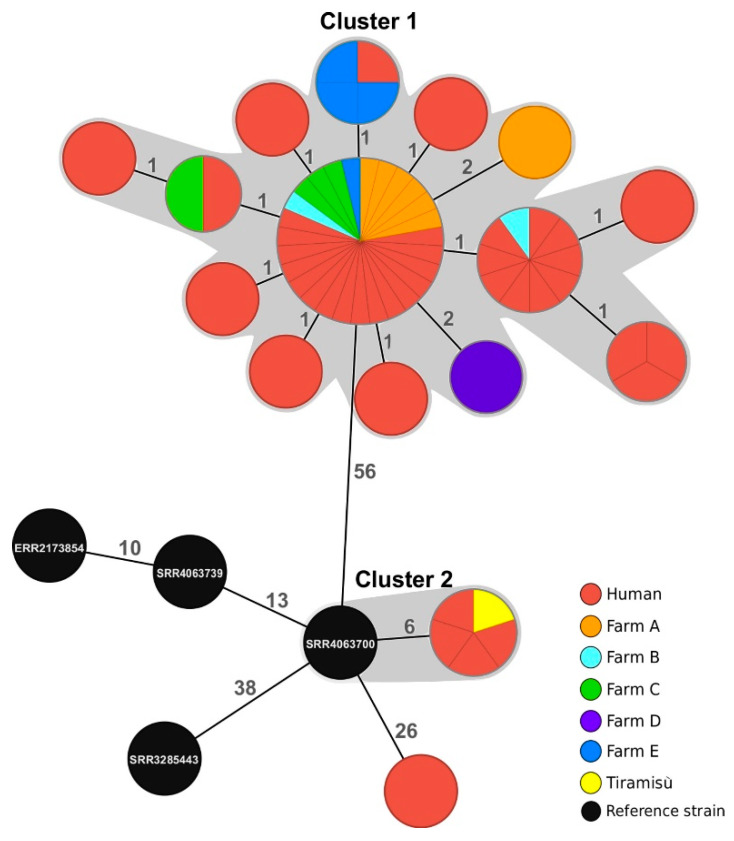
Minimum spanning tree generated for 65 samples using core-genome Multilocus Sequence Typing (cgMLST). MLST was based on pairwise comparison of 3002 genes. Branch lengths correspond to the number of discriminating loci Clusters of related genotypes, defined as profiles located within a single-linkage distance of seven loci, and are highlighted in grey. Nodes depicted in black correspond to the four isolates used by the European Food Standards Agency (EFSA) and the European Centre for Disease Prevention and Control (ECDC) to define confirmed outbreak cases in the multi-country outbreak linked to Polish eggs.

Core genome analysis revealed the presence of two separate clusters of related genotypes that differed by no more than seven alleles from one another (Figure 2). The larger one, Cluster 1, grouped all strains collected from the farms and from the majority of human cases, suggesting that for the majority of the patients from Abruzzi, gastroenteritis was acquired from local products. The single-linkage distance within this group of strains did not exceed tow loci, and the maximum pairwise distance detected was five genes. 

Since five of the isolates were assigned to the MLVA profile connected to the multi-national outbreak in Europe, in our analysis, we additionally included four strains of *S.* Enteritidis defined as confirmed outbreak strains [19]. Sample SRR4063700 clustered together with the tiramisù strain and with four human isolates. The five Italian strains shared an identical core genome MLST profile and were six loci distant from the reference isolate, confirming their close genetic relatedness. The only isolate that did not belong to any cgMLST cluster was obtained from a human case (2018-TE-5788-1-1) and was located 26 core genes away from the reference strain SRR4063700. 

In order to further confirm genetic relatedness of the isolates within the two detected cgMLST clusters, we performed SNP analysis and assigned SNP addresses to all the isolates in our dataset (Table 1). All isolates in Cluster 1 shared the SNP address 2.2.2.2.2.2.%, i.e., each of the strains was located within the single-linkage distance of no more than five SNPs from the closest neighbouring isolate, which confirmed that they were part of the same outbreak in Abruzzi. The maximum pairwise distance found in this cluster was eight SNPs. 

The four human isolates and the strain isolated from tiramisù shared the same SNP address (2.7.7.20.20.20.20), confirming that the dessert was the likely source of food poisoning (Table 1). The reference strain SRR4063700, which was placed in the same cgMLST cluster, was assigned an SNP address that was identical up to the 25 SNP threshold level (2.7.7.20.52.52.52), and therefore the Italian samples did not fit the definition of “confirmed outbreak case” in the multi-country *S.* Enteritidis outbreak. The distance between SRR4063700 and the Italian isolates was 13 SNPs. The human isolate 2018-TE-5788-1-1 differed by 52 SNPs from the closest neighbour (SRR4063700).

**Figure 3:**
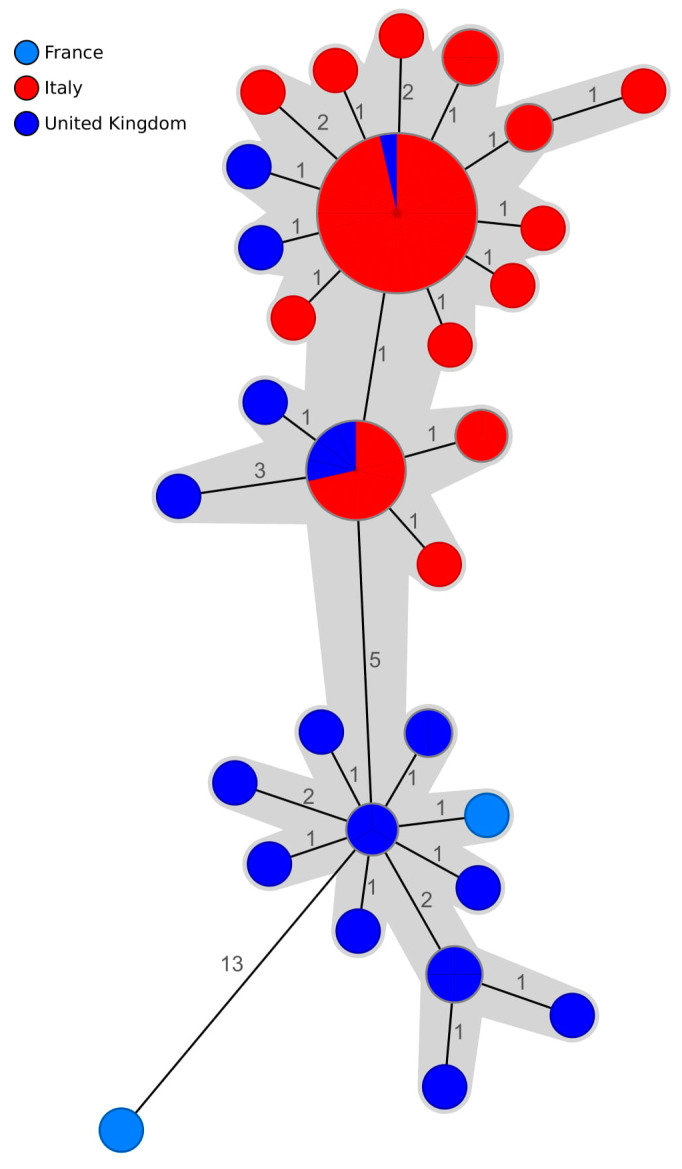
Minimum spanning tree of a subset of *S.* Enteritidis isolates based on cgMLST profile comparison. The tree was generated in Ridom SeqSphere+ using an integrated *Salmonella enterica* cgMLST scheme (3002 target genes). The Italian isolates sequenced in this study are depicted in red, and the isolates from UK and France obtained from EnteroBase are shown in dark blue and light blue, respectively. The branch labels correspond to the number of different alleles between pairs of cgMLST profiles. The complex of genotypes located within a single-linkage distance of seven loci is highlighted in grey.

Lastly, to establish whether the isolates in Cluster 1 were genetically connected to the other *S.* Enteritidis strains in Italy and in Europe, we performed cgMLST analysis and comparison of Cluster 1 isolates and *Salmonella* strains deposited in EnteroBase. It was possible to retrieve only 32 Italian *S.* Enteritidis strains, most of which had been collected in 2016. Comparison of cgMLST profiles showed 59 allele differences between these strains and a representative strain from our study (2018-TE-19130-1-1; Figure S1). We then expanded our analysis to include all isolates of *S.* Enteritidis ST-11 collected in Europe in 2018 (Figure S2). Our search retrieved 4585 entries, and we limited our analysis to isolates that were grouped within a difference threshold of 50 loci from 2018-TE-19130-1-1 (Figure 3). We identified a cluster of genetically related profiles that included isolates collected in UK and France from humans, two of which shared the same cgMLST type as the isolate from our study. Comparison of the 27 isolates from UK and France and the Abruzzi Cluster 1 strains showed that all, except for one French isolate, grouped within the maximum single-linkage distance of five alleles. 

## 3. Discussion

According to our data, at least two different outbreaks of gastroenteritis caused by *S.* Enteritidis occurred during 2018 in the Abruzzi Italian region. The first one, which affected a larger number of patients, was caused by local strains and was traced back to several farms found in the region. All strains obtained from farm environment and from poultry had very closely related genotypes, suggesting that the animals could have been obtained from the same livestock producer or that the farms shared a common food supplier that had provided a contaminated product, spreading the infection in an isolated geographic region. A core genome MLST analysis of publicly available Italian *S.* Enteritidis strains did not show significant genetic similarity between the isolates from our study and the strains accessed through EnteroBase [20]. Unfortunately, the public dataset included only entries from 2016 or older and therefore did not provide any additional information about the prevalence of Cluster 1 clone in Italy. It would therefore be interesting to widen the analysis by sequencing isolates from other regions in order to understand if cgMLST Cluster 1 was generally highly prevalent in Italy in 2018 or if it was confined to specific geographic locations. 

The circulation of the same clone between farms and humans can be explained by the time gap that occurred between the distribution of the contaminated egg lots to stores and the assessment and notification of *Salmonella* contamination by the health authorities. The Italian law imposes an immediate block of the eggs’ sale when an outbreak is confirmed in a farm. From the time of notification, the eggs are no longer intended for direct consumption and have to be recalled. However, until an outbreak is detected, contaminated egg products can circulate in the market, often for several months, and their identification depends on the frequency of controls in farms. Indeed, several batches of eggs entered the market in 2018 and were later recalled by the Italian Ministry of Health because of confirmed *Salmonella* contamination in a large laying hen farm in Abruzzi [21]. It is therefore possible that contaminated eggs were purchased and used, causing multiple cases of salmonellosis in the region. Moreover, uncertified eggs obtained “straight from the farm” or from the backyard chickens may pose additional risk for infection.

The circulation of the same clone within multiple farms could be explained by the purchase of laying hens from the same producer. At the end of each production cycle, egg-producing poultry farms present in the Abruzzi region purchase hens from the same geographical area to be brought together for the new cycle. It is therefore possible that the hens purchased by these companies and destined for the repopulation of several farms were all contaminated with *Salmonella*. This could explain the circulation of the same clone between unrelated local farms in such a short time period. 

We were not able to assign one human isolate to any WGS clusters, but the closest genotype, distant by 52 SNPs, was the reference strain SRR4063700, suggesting that the source of infection was not linked to the poultry or eggs produced in Abruzzi. It has to be highlighted that, as currently there are no recommendations by the EFSA or ECDC for the specific cgMLST cut-off to be used for inclusion of an isolate in the outbreak cluster, in our analysis we used the default setting provided by the software. In the case of Cluster 1, the SNP analysis confirmed the inclusion of all strains in the outbreak complex.

We extended our cgMLST analysis further to include *S.* Enteritidis isolated in Europe in 2018 and we identified a cluster of strains from UK and France associated with human infections. As no genetically related isolates from animal or environmental sources were found in Europe, it is difficult to determine if these cases were related to contaminated eggs or food products imported from Italy or whether some poultry farms in UK and France also harboured the outbreak clone. Italy is one of the first five biggest egg producers in the EU [22], and therefore it is possible that *Salmonella*-contaminated products originating in Italy were released into the EU market. Indeed, in the last decade, five EFSA reports related to multi-country outbreaks of salmonellosis, including outbreaks originating from German and Polish egg producers, have been published [9,20,23].

The second cgMLST cluster, shared by four human and one tiramisù isolate was not present in the local farms included in our dataset. In fact, close genetic relatedness revealed by WGS analysis pointed at a foreign origin of the strains, which shared the same cgMLST complex with the reference sequence from the multinational outbreak of *S.* Enteritidis. Until this date, 26 confirmed cases of salmonellosis linked to Polish eggs have been reported in Italy by the EFSA and ECDC, 7 of which occurred after 2017 [19]. This shows that either contaminated eggs were sold in Italy (and possibly also used in the preparation of the tiramisù) or laying hens were imported from the production farm contaminated with *S.* Enteritidis. While we did not detect any related strains in Abruzzi farms, the examination of isolates involved in outbreaks in different regions in Italy could shed more light on the origin of *Salmonella*-positive eggs in the Italian market.

As indicated by the ESFA and ECDC, we assigned SNP addresses to all isolates from this study. Italian strains in Cluster 2 did not fit the definition of confirmed outbreak case when compared with the most closely related reference strain. However, the SNP address is generated based on single-linkage distance and therefore it relies on the availability of a complete set of sequences to reflect the true diversity of the strains within a large outbreak that has been active for several years. The Italian isolates, both human and from the dessert, shared exactly the same SNP address, but the reference strain was distant by 13 SNPs. In a recent work, Pijnacker et al. (2019) [24] analysed *S.* Enteritidis strains collected during the multi-country outbreak between 2015 and 2018 and divided them into two WGS clusters. They found that in WGS Cluster 2 (which would include isolate SRR4063700 used in our study), the maximum SNP distance between any two isolates was 37 SNPs. The distance of 13 SNPs, in the case of our Cluster 2, therefore fits within this threshold, and we strongly believe that these five isolates could be included in those that caused the multi-country outbreak. 

In their report, the EFSA and ECDC have strongly encouraged the countries affected by the outbreak of *S.* Enteritidis to perform WGS analysis of food isolates collected by individual laboratories. Currently, it might be cumbersome to filter out unrelated data from public repositories, and thus laboratories that lack computing and storage resources may discouraged from performing cluster analysis using the SNP address approach. An international effort to make sequencing data publicly accessible and recognizable as derived from that specific outbreak is required to enable individual laboratories to take an active part in typing and detecting epidemiological clusters. Moreover, it would be useful to include cgMLST typing as a more accessible alternative to SNP analysis, as previously shown by the EFSA and ECDC, which used it to define cases in a multi-country outbreak of *Salmonella* Agona infections [25]. Indeed, a previous study by Pearce and colleagues (2018) demonstrated that cgMLST clustering using the EnteroBase cgMLST typing schema for *S. enterica* was congruent with SNP-based analysis, and the gene-by-gene approach provided high resolution and was easy to standardize [26].

Our study showed that the majority of human cases of foodborne gastroenteritis caused by *S.* Enteritidis in 2018 in Abruzzi were caused by strains circulating in local farms. Increased surveillance and repeated controls in poultry farms and egg production facilities are therefore essential to eliminate the infection from the region. Moreover, while the outbreak in the EU is ongoing, the WGS analysis of collected isolates should be performed routinely to confirm cases linked to the expanding European cluster. To date, the exact source of Polish eggs contamination is unknown, and therefore prompt identification and confirmation of related cases remain critical for the epidemiological investigation of the outbreak in Europe. 

## 4. Materials and Methods 

### 4.1. Dataset

As part of the National Reference Laboratory’s activity for monitoring *Salmonella* in the Abruzzi region, 61 *S.* Enteritidis strains were analysed by WGS in 2018. The strains were isolated from human faecal samples (42), tiramisù (1) and six poultry farms (18) and included 13 chicken cloacal swabs and 6 environmental swabs. Additionally, sequencing reads of four *S.* Enteritidis strains previously used for the definition of an outbreak case in the multi-country outbreak of *S.* Enteritidis linked to Polish eggs were obtained from the NCBI Sequence Reads Repository (SRA accessions: SRR3285443, SRR4063700, ERR2173854 and SRR4063739) [19]. 

### 4.2. Salmonella Culture and Serotyping

The isolates were cultured overnight at 37 °C in Rambach agar. The isolates were serotyped with commercial antisera (Statens Serum Institut, Copenhagen, Denmark) according to the Kauffmann–White scheme, by slide agglutination [27,28].

Antimicrobial susceptibility test was performed by the microdilution method using the Sensititre automated system with TES (Thermo Fisher, Italia) and the Sensititre EUVSEC (Thermo Fisher, Italia) panel of 14 antimicrobials: ampicillin (1–64 µg/mL), azithromycin (2–64 µg/mL), cefotaxime (0.25–4 µg/mL), ceftazidime (0.5–8 µg/mL), chloramphenicol (8–128 µg/mL), ciprofloxacin (0.015–8 µg/mL), colistin (1–16 µg/mL), gentamicin (0.5–32 µg/mL), meropenem (0.03–16 µg/mL), nalidixic Acid (4–128 µg/mL), sulfamethoxazole (8–1024 µg/mL), tetracycline (2–64 µg/mL), tigecycline (0.25–8 µg/mL) e timethoprim (0.25–32 µg/mL).

### 4.3. Multilocus Variable-Number Tandem Repeat Analysis 

Bacterial DNA was extracted with the Maxwell® 16 Tissue DNA Purification Kit using the Maxwell® 16 instrument (Promega, Madison, WI, USA) according to the manufacturer’s instructions. To assign specific alleles, DNA was amplified by multiplex PCR using primers specific for each VNTR locus in a 5-locus MLVA scheme (SENTR7, SENTR5, SENTR6, SENTR4 and SE-3), as described before [29]. The amplicons were then separated by capillary electrophoresis using an ABI 3500 instrument with POP 7 polymer, and the allele types were assigned using GeneMapper 4.1 (Applied Biosystems, Carlsbad, CA). MLVA profiles were analysed using the goeBURST algorithm implemented in PHYLOViZ, version 2.0 [30].

### 4.4. Next-Generation Sequencing

Th genomic DNA of 61 strains of *S.* Enteritidis was sequenced using the Illumina NextSeq 500 platform with 150 bp paired-end reads. Briefly, the DNA was quantified using Qubit fluorometer (QubitTM DNA HS assay; Life Technologies, Thermo Fisher Scientific Inc., Waltham, MA, USA), and the sequence libraries were prepared using the Nextera XT library preparation kit (Illumina Inc., San Diego, CA, USA), following the standard protocol. The sequence reads were trimmed using Trimmomatic v0.36, and the scaffolds were assembled with SPAdes v3.11.1 [31,32].

The read sequences were stored in the Sequence Read Archive of the National Center for Biotechnology Information (NCBI) under the BioProject accession number PRJNA612025.

### 4.5. In Silico MLVA Analysis

The MLVA profiles of four samples obtained from the SRA database were determined directly from the scaffolds. The reads were assembled as described above, and MLVA profiles were generated using the MLVA In Silico Typing Resource for Salmonella Strains (MISTReSS, https://github.com/Papos92/MISTReSS#mistress-mlva-in-silico-typing-resource-for-salmonella-strains) using the primer set included in the MISTReSS package. 

### 4.6. MLST and Core-Genome MLST Analysis

Core-genome allele assignment and analysis were performed in Ridom SeqSphere+ software, version 6.0.2 (Ridom GmbH, Münster, Germany), using the *S. enterica* core-genome MLST task template version 2.0, which uses the same 3002 loci as the EnteroBase (http://enterobase.warwick.ac.uk) *S.* *enterica* cgMLST v2. Default settings were applied for allele calling and cluster detection (cluster cut-off ≤7 loci). A multiple spanning tree (MST) was generated by comparison of cgMLST loci between pairs of isolates. The missing values were ignored in distance calculation. All sequences were additionally typed using the Achtman Salmonella 7 locus Multilocus Sequence Typing scheme available at http://enterobase.warwick.ac.uk/species/index/senterica, accessible through Ridom SeqSphere+.

Additional analysis was performed using the cgMLST V2 scheme in EnteroBase [33,34]. Two searches of the *Salmonella* database were performed (database accessed on 20 April 2020). To generate the first dataset, *S.* Enteritidis entries were filtered to include strains isolated in Italy only. The second dataset included *S.* Enteritidis strains filtered to retain the European isolates from 2018 with assigned ST-11 and assemblies with N50 > 50,000. Both datasets were used to generate MSTs based on the cgMLST profiles using GrapeTree tool [35]. 

### 4.7. SNP Address Assignment

SNP addresses were assigned to S. Enteritidis strains using SnapperDB, which utilizes PHEnix pipeline (https://github.com/phe-bioinformatics/PHEnix) [36].

Briefly, trimmed reads were aligned to *S.* *enterica* subsp. enterica serovar Enteritidis str. P125109 reference (NCBI accession GCA_000009505.1), using BWA-MEM version 0.712-r1039, and the SNPs were called using GATK 3.9. The VCF was parsed using default parameters (depth_cutoff = 10, mq_cutoff = 30, ad_cutoff = 0.9) [37,38].

## Figures and Tables

**Table 1:** List of *Salmonella* Enteritidis isolates used in the study. SNP, Single-Nucleotide Polymorphism.

Sample ID	Isolation Source	Farm Name	MLVA	cgMLST Profile	cgMLST Cluster	SNP Address
2018-AZ-4484-1-4	Chicken	Farm A	2-10-7-3-2	1915	Cluster 1	2.2.2.2.2.2.2
2018-AZ-4706-1-2	Chicken	Farm C	2-10-7-3-2	1915	Cluster 1	2.2.2.2.2.2.8
2018-AZ-4766-1-2	Chicken	Farm A	2-10-7-3-2	1915	Cluster 1	2.2.2.2.2.2.2
2018-AZ-4767-1-2	Chicken	Farm A	2-10-7-3-2	3078		2.2.2.2.2.2.2
2018-AZ-5011-1-3	Chicken	Farm A	2-10-7-3-2	3071	Cluster 2	2.2.2.2.2.2.34
2018-AZ-5621-1-12	Farm environment	Farm C	2-10-7-3-2	1915	Cluster 1	2.2.2.2.2.2.24
2018-AZ-5621-1-13	Farm environment	Farm C	2-10-7-3-2	1915	Cluster 1	2.2.2.2.2.2.23
2018-AZ-5715-1-3	Chicken	Farm E	2-10-7-3-2	1915	Cluster 1	2.2.2.2.2.2.2
2018-AZ-5718-1-2	Chicken	Farm E	1-10-7-3-2	1915	Cluster 1	2.2.2.2.2.2.9
2018-AZ-5718-2-2	Farm environment	Farm E	1-10-7-3-2	1915	Cluster 1	2.2.2.2.2.2.9
2018-AZ-6087-1-21	Chicken	Farm D	2-11-7-3-2	1915	Cluster 1	2.2.2.2.2.2.25
2018-AZ-6266-1-2	Chicken	Farm C	2-10-7-3-2	1915	Cluster 1	2.2.2.2.2.2.2
2018-AZ-7226-1-2	Chicken	Farm E	1-10-7-3-2	1915	Cluster 1	2.2.2.2.2.2.51
2018-CB-3223-1-7	Tiramisù		2-9-7-3-2	3071	Cluster 2	2.7.7.20.20.20.20
2018-CB-3513-1-11	Farm environment	Farm B	2-10-7-3-2	1915	Cluster 1	2.2.2.2.2.2.2
2018-CB-3513-1-16	Farm environment	Farm B	2-10-7-3-2	1915	Cluster 1	2.2.2.2.2.2.2
2018-PE-6339-1-11	Chicken	Farm A	2-10-7-3-2	1915	Cluster 1	2.2.2.2.2.2.2
2018-TE-12516-1-1	Human		2-10-7-3-2	1915	Cluster 1	2.2.2.2.2.2.66
2018-TE-12881-1-1	Human		2-10-7-3-2	1915	Cluster 1	2.2.2.2.2.2.2
2018-TE-14038-1-1	Human		2-10-7-3-2	1915	Cluster 1	2.2.2.2.2.2.49
2018-TE-15229-1-1	Chicken	Farm A	2-10-7-3-2	1915	Cluster 1	2.2.2.2.2.2.2
2018-TE-16067-1-1	Human		2-10-7-3-2	1915	Cluster 1	2.2.2.2.2.2.39
2018-TE-16075-1-1	Human		2-10-7-3-2	1915	Cluster 1	2.2.2.2.2.2.2
2018-TE-17020-1-1	Human		2-10-7-3-2	1915	Cluster 1	2.2.2.2.2.2.2
2018-TE-17030-1-1	Human		2-10-7-3-2	1915	Cluster 1	2.2.2.2.2.2.2
2018-TE-17605-1-1	Human		2-10-7-3-2	1915	Cluster 1	2.2.2.2.2.2.42
2018-TE-19012-1-1	Farm environment	Farm A	2-10-7-3-2	1915	Cluster 1	2.2.2.2.2.2.31
2018-TE-19126-1-1	Human		2-11-7-3-2	1915	Cluster 1	2.2.2.2.2.2.16
2018-TE-19129-1-1	Human		2-11-7-3-2	1915	Cluster 1	2.2.2.2.2.2.16
2018-TE-19130-1-1	Human		2-10-7-3-2	1915	Cluster 1	2.2.2.2.2.2.2
2018-TE-19418-1-1	Human		2-10-7-3-2	1915	Cluster 1	2.2.2.2.2.2.2
2018-TE-20273-1-2	Human		2-10-7-3-2	1915	Cluster 1	2.2.2.2.2.2.45
2018-TE-20299-1-1	Human		2-10-7-3-2	1915	Cluster 1	2.2.2.2.2.2.2
2018-TE-20303-1-1	Human		2-10-7-3-2	1915	Cluster 1	2.2.2.2.2.2.17
2018-TE-20304-1-1	Human		2-10-7-3-2	1915	Cluster 1	2.2.2.2.2.2.8
2018-TE-20305-1-1	Human		2-10-7-3-2	1915	Cluster 1	2.2.2.2.2.2.2
2018-TE-22807-1-1	Human		2-10-7-3-2	1915	Cluster 1	2.2.2.2.2.2.42
2018-TE-24761-1-10	Human		2-11-7-3-2	1915	Cluster 1	2.2.2.2.2.2.59
2018-TE-24761-1-11	Human		2-10-7-3-2	1915	Cluster 1	2.2.2.2.2.2.45
2018-TE-24761-1-13	Human		2-10-7-3-2	1915	Cluster 1	2.2.2.2.2.2.45
2018-TE-24761-1-3	Human		2-10-7-3-2	1915	Cluster 1	2.2.2.2.2.2.2
2018-TE-24761-1-4	Human		2-11-7-3-2	1915	Cluster 1	2.2.2.2.2.2.19
2018-TE-24761-1-5	Human		2-11-7-3-2	1915	Cluster 1	2.2.2.2.2.2.11
2018-TE-24761-1-9	Human		2-10-7-3-2	1915	Cluster 1	2.2.2.2.2.2.30
2018-TE-26653-1-1	Human		2-10-7-3-2	1915	Cluster 1	2.2.2.2.2.2.2
2018-TE-26653-1-2	Human		2-11-7-3-2	1915	Cluster 1	2.2.2.2.2.2.11
2018-TE-26653-1-3	Human		2-11-7-3-2	1915	Cluster 1	2.2.2.2.2.2.11
2018-TE-26653-1-6	Human		2-11-7-3-2	1915	Cluster 1	2.2.2.2.2.2.11
2018-TE-26685-1-1	Human		2-10-7-3-2	1915	Cluster 1	2.2.2.2.2.2.26
2018-TE-26685-1-2	Human		2-10-7-3-2	1915	Cluster 1	2.2.2.2.2.2.26
2018-TE-26685-1-3	Human		2-10-7-3-2	1915	Cluster 1	2.2.2.2.2.2.26
2018-TE-26685-1-4	Human		2-9-7-3-2	3071	Cluster 2	2.7.7.20.20.20.20
2018-TE-26685-1-5	Human		2-9-7-3-2	3071	Cluster 2	2.7.7.20.20.20.20
2018-TE-26685-1-6	Human		2-9-7-3-2	3071	Cluster 2	2.7.7.20.20.20.20
2018-TE-26685-1-7	Human		2-9-7-3-2	3071	Cluster 2	2.7.7.20.20.20.20
2018-TE-5787-1-1	Human		2-10-7-3-2	1915	Cluster 1	2.2.2.2.2.2.57
2018-TE-5788-1-1	Human		2-11-7-3-2	1915		2.7.7.28.28.28.28
2018-TE-7355-1-1	Human		2-10-7-3-2	1915	Cluster 1	2.2.2.2.2.2.2
2018-TE-8898-1-1	Human		2-10-7-3-2	1915	Cluster 1	2.2.2.2.2.2.67
2018-TE-8904-1-1	Human		2-10-7-3-2	1915	Cluster 1	2.2.2.2.2.2.10
2018-TE-9213-1-1	Human		2-10-7-3-2	1915	Cluster 1	2.2.2.2.2.2.6
ERR2173854	Chicken		2-10-7-3-2	3077		2.7.7.7.7.7.7
SRR3285443	Human		2-9-7-3-2	384		2.7.15.15.15.15.15
SRR4063700	Human		2-9-7-3-2	387	Cluster 2	2.7.7.20.52.52.52
SRR4063739	Human		2-10-8-3-2	546		2.7.7.7.21.21.21

## Supplementary Materials

The following are available online at https://www.mdpi.com/2076-0817/9/5/349/s1, Figure S1: Grape tree MSTree (V2) of Italian *S.* Enteritidis isolates based on cgMLST V2 analysis in EnteroBase. The representative strain from this study is depicted in red and compared with publicly available strains. Branch distances correspond to the number of different core genes between pairs of genotypes. Figure S2. GrapeTree based on cgMLST of *S.* Enteritidis strains isolated in 2018 in Europe. MSTree (V2) was generated using cgMLST V2 in EnteroBase. The isolates differing by no more than 50 alleles from the Italian strain from our study (depicted in red) are shown. The cluster of genomes containing the Italian strain is highlighted in yellow.
