# Supplementary material for: Genomic Comparison of Salmonella Enteritidis Strains Isolated from Laying Hens and Humans in the Abruzzi Region during 2018
Source: Pathogens. 2020 May 5;9(5):349. doi: 10.3390/pathogens9050349 (PMC7281747; doi:10.3390/pathogens9050349)
Supplement: Supplementary file 1 [file pathogens-09-00349-s001.zip › pathogens-773500-supplementary/Supplementary figure.docx]

**Supplementary figures**


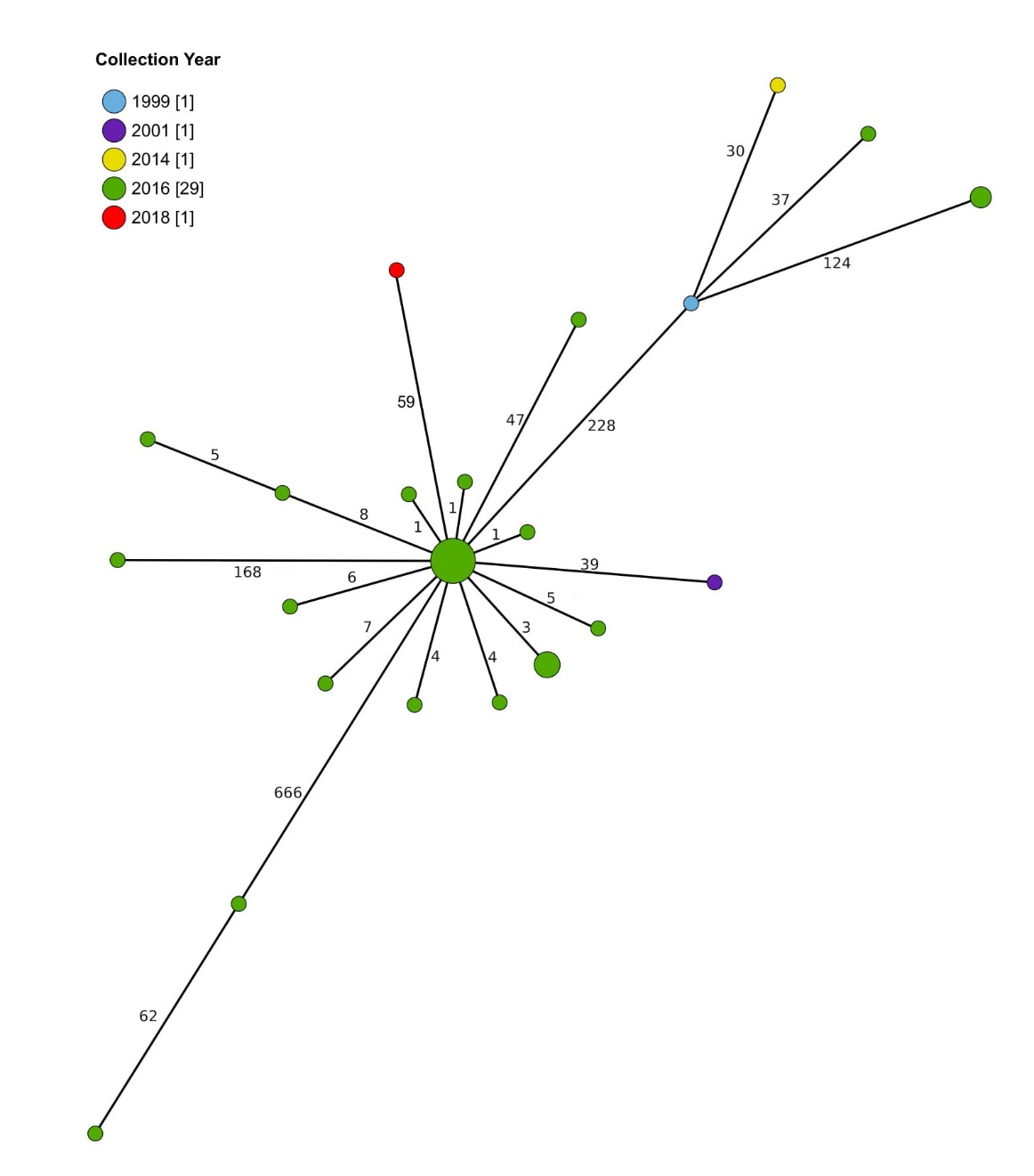


**Figure S1.** Grape tree MSTree (V2) of Italian *S.* Enteritidis isolates based on cgMLST V2 analysis in EnteroBase. The representative strain from this study is depicted in red and compared with publicly available strains. Branch distances correspond to the number of different core genes between pairs of genotypes.


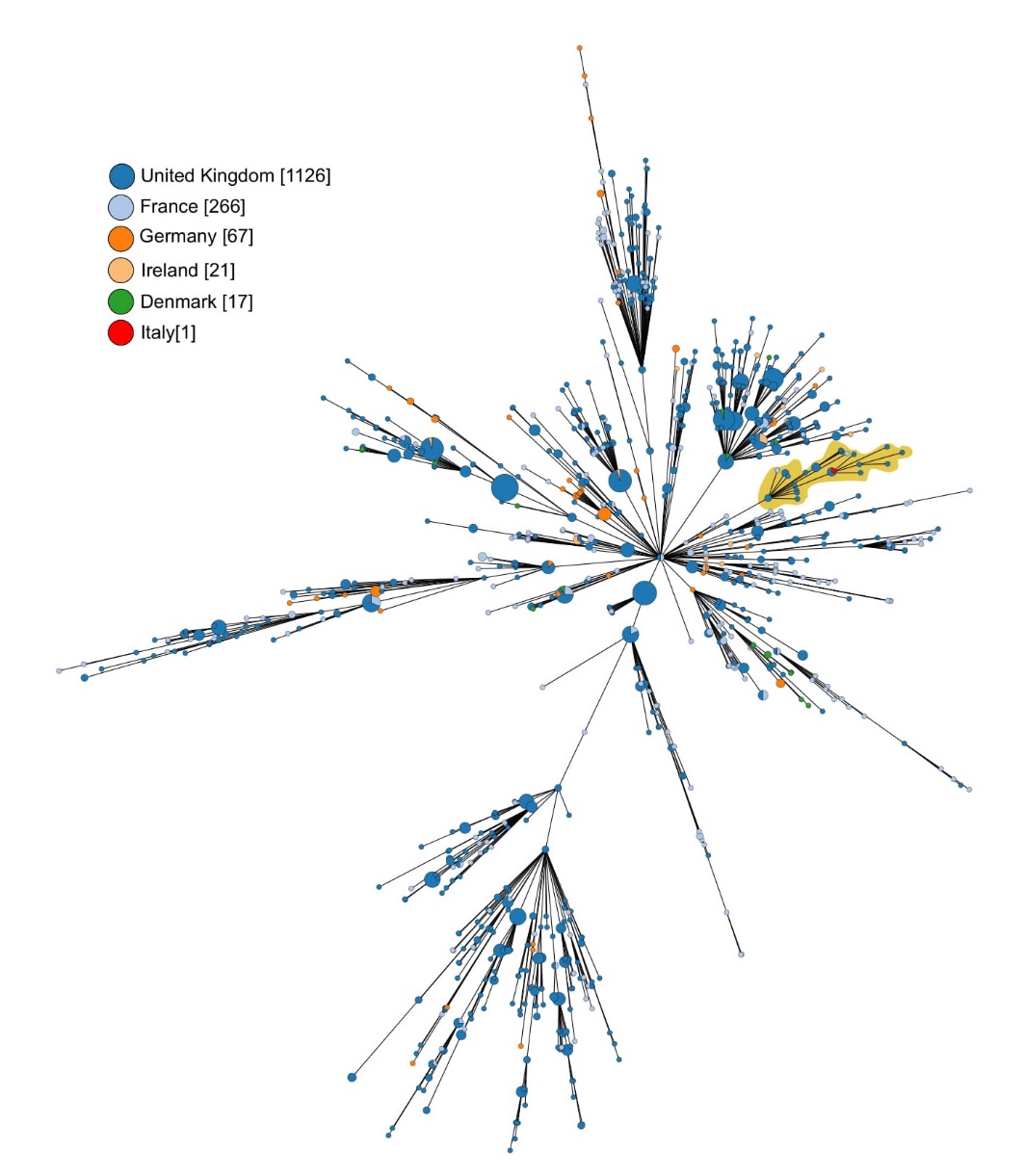


Figure S2. GrapeTree based on cgMLST of *S.* Enteritidis strains isolated in 2018 in Europe. MSTree (V2) was generated using cgMLST V2 in EnteroBase. The isolates differing by no more than 50 alleles from Italian strain from our study (depicted in red) are shown. Cluster of genomes containing Italian strain is highlighted in yellow.
