# Supplementary figures and images for: Genomic Comparison of Salmonella Enteritidis Strains Isolated from Laying Hens and Humans in the Abruzzi Region during 2018
Source: Pathogens. 2020 May 5;9(5):349. doi: 10.3390/pathogens9050349 (PMC7281747; doi:10.3390/pathogens9050349)

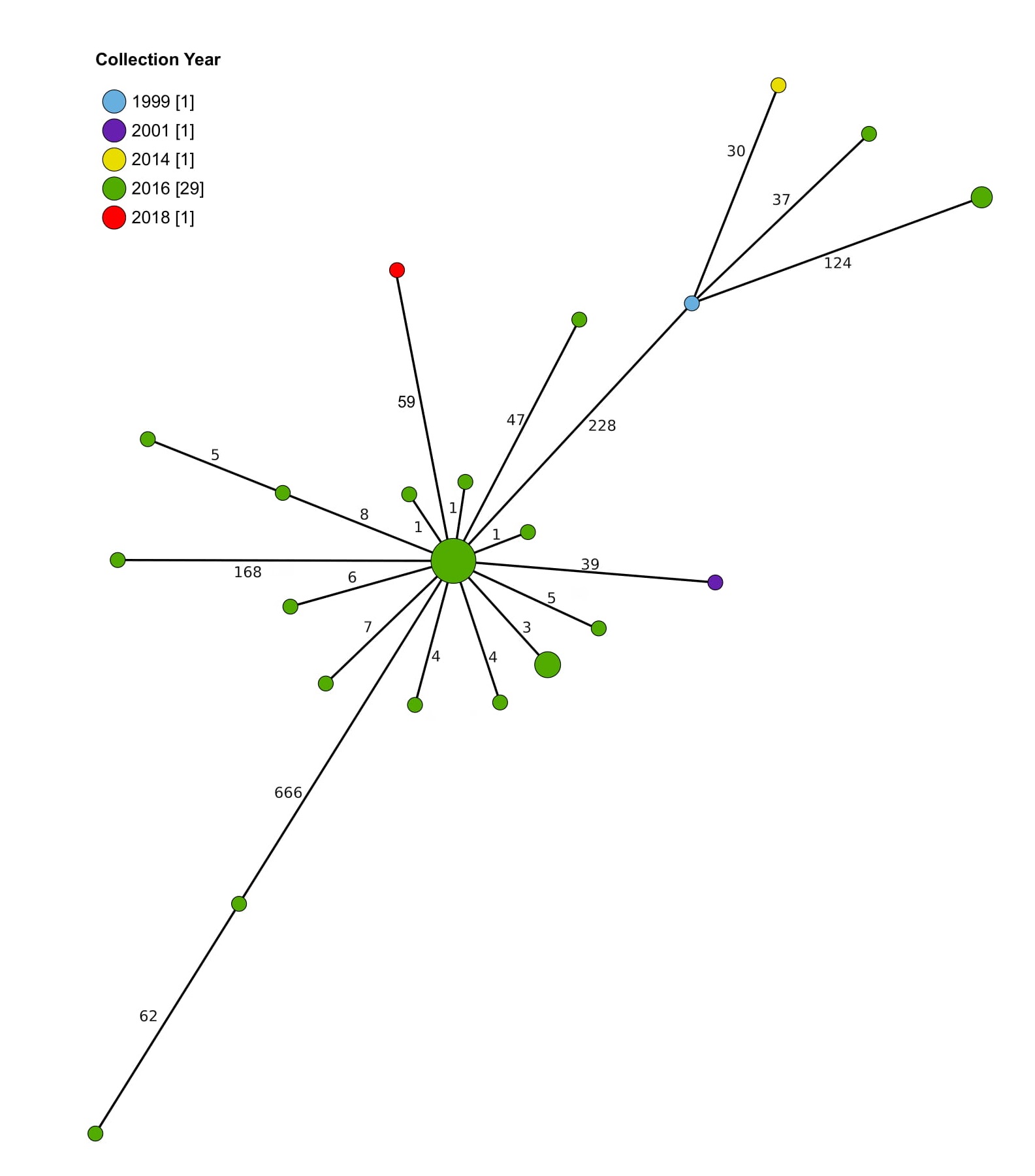

Supplement: Supplementary file 1 [file pathogens-09-00349-s001.zip › pathogens-773500-supplementary/Figure S1.jpg]

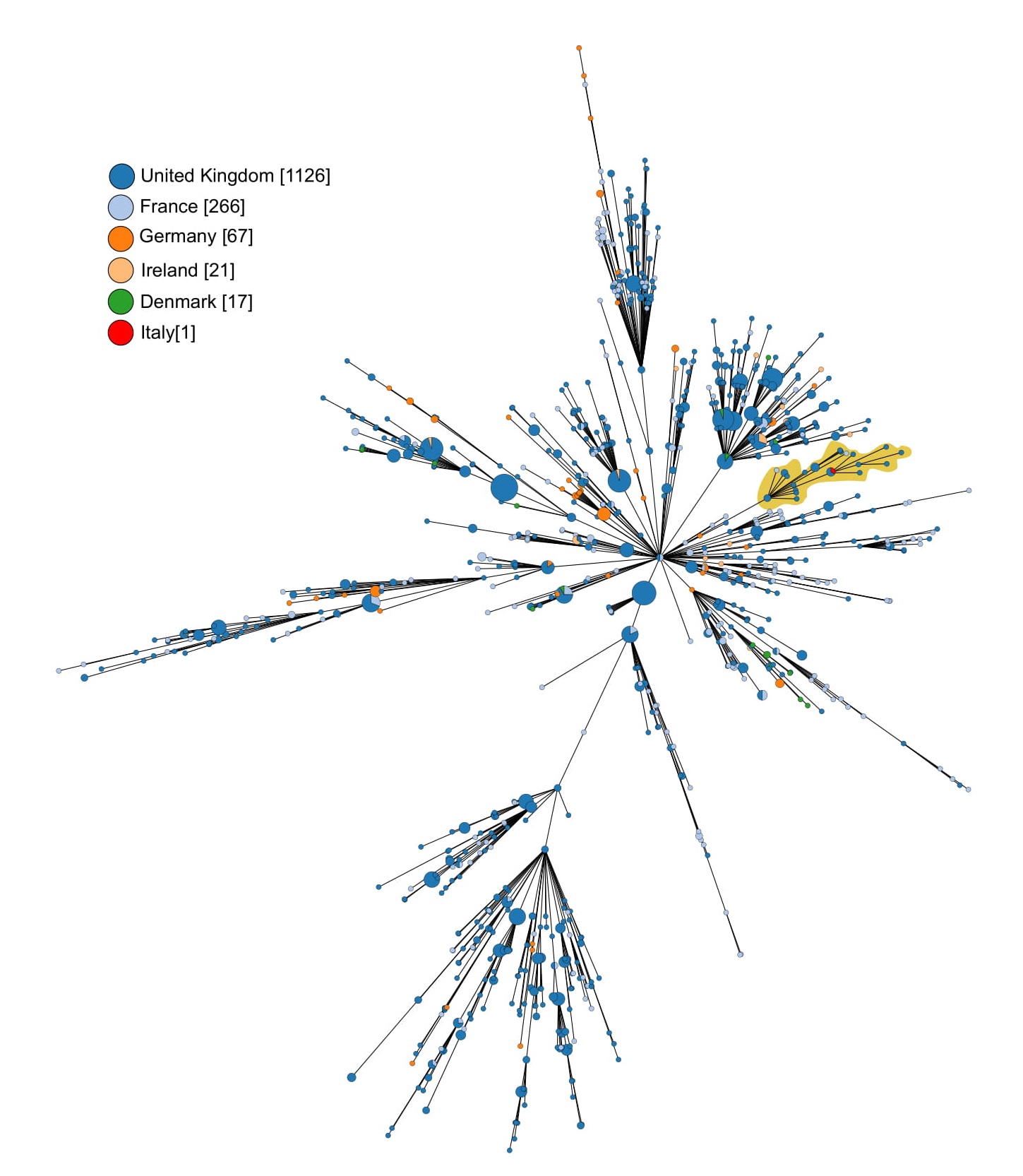

Supplement: Supplementary file 1 [file pathogens-09-00349-s001.zip › pathogens-773500-supplementary/Figure S2.jpg]
